# Supplementary material for: Rotavirus Calcium Dysregulation Manifests as Dynamic Calcium Signaling in the Cytoplasm and Endoplasmic Reticulum
Source: Sci Rep. 2019 Jul 25;9:10822. doi: 10.1038/s41598-019-46856-8 (PMC6658527; doi:10.1038/s41598-019-46856-8)
Supplement: Supplementary file 1 — Supplementary Figures and Video Legends [file 41598_2019_46856_MOESM1_ESM.pdf]

Rotavirus Calcium Dysregulation Manifests as Dynamic Calcium Signaling in the Cytoplasm and Endoplasmic Reticulum

Alexandra L. Chang-Graham[1], Jacob L. Perry[1], Alicia C. Strtak[1], Nina K. Ramachandran[1], Jeanette M. Criglar[1], Asha A. Philip[3], John T. Patton[3], Mary K. Estes[1,2], and Joseph M. Hyser[1]*

[1]Alkek Center for Metagenomic and Microbiome Research; Department of Molecular Virology and Microbiology; [2]Department of Medicine, Gastroenterology and Hepatology, Baylor College of Medicine, Houston, TX 77303. [3]Department of Biology, Indiana University, Bloomington, IN 47405

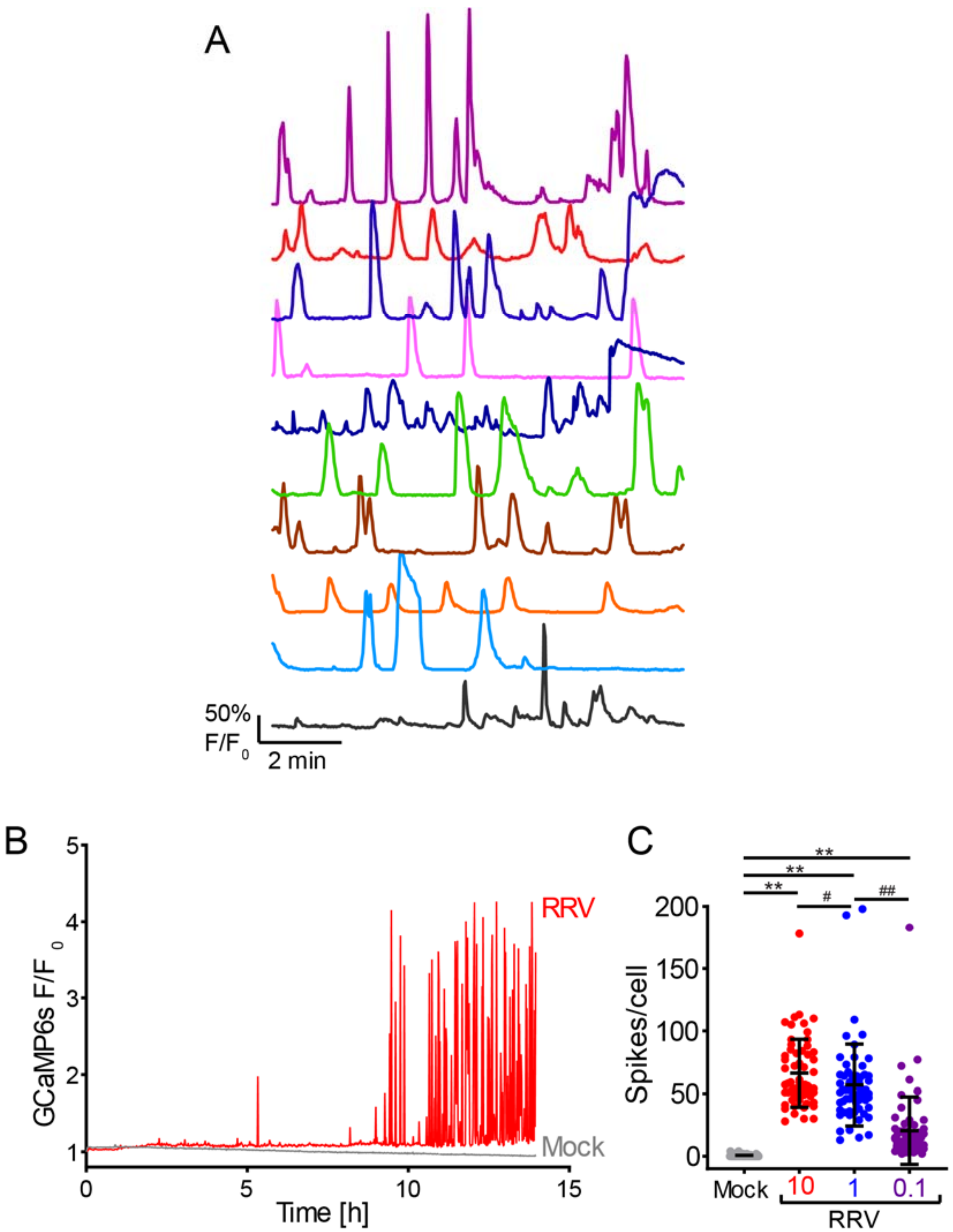

Supplementary Figure 1. A. Representative Ca^{2+} traces (F/F_0) from 10 MA104-GCaMP5G cells infected with SA114F at MOI 10. Cells were imaged for 10 minutes at ~7 hpi with an image acquisition frequency of 1 image/1.5 seconds. These data correspond to Supplementary Video 3 online. **B.** Representative single-cell traces of relative GCaMP5G fluorescence (F/F_0) from cells mock (grey) or RV infected by RRV at MOI 10 (red). **C.** Number of Ca^{2+} spikes (F/F_0 > 5%) from mock or RV-infected cells that were infected with RRV at MOI 10, 1 or 0.1. N= 60 cells per condition. $^{**}p<0.01$ compared to Mock; $^{\#}p<0.05$ comparing MOI 10 to MOI 1; $^{\#\#}p<0.01$ comparing MOI 1 and MOI 0.1.

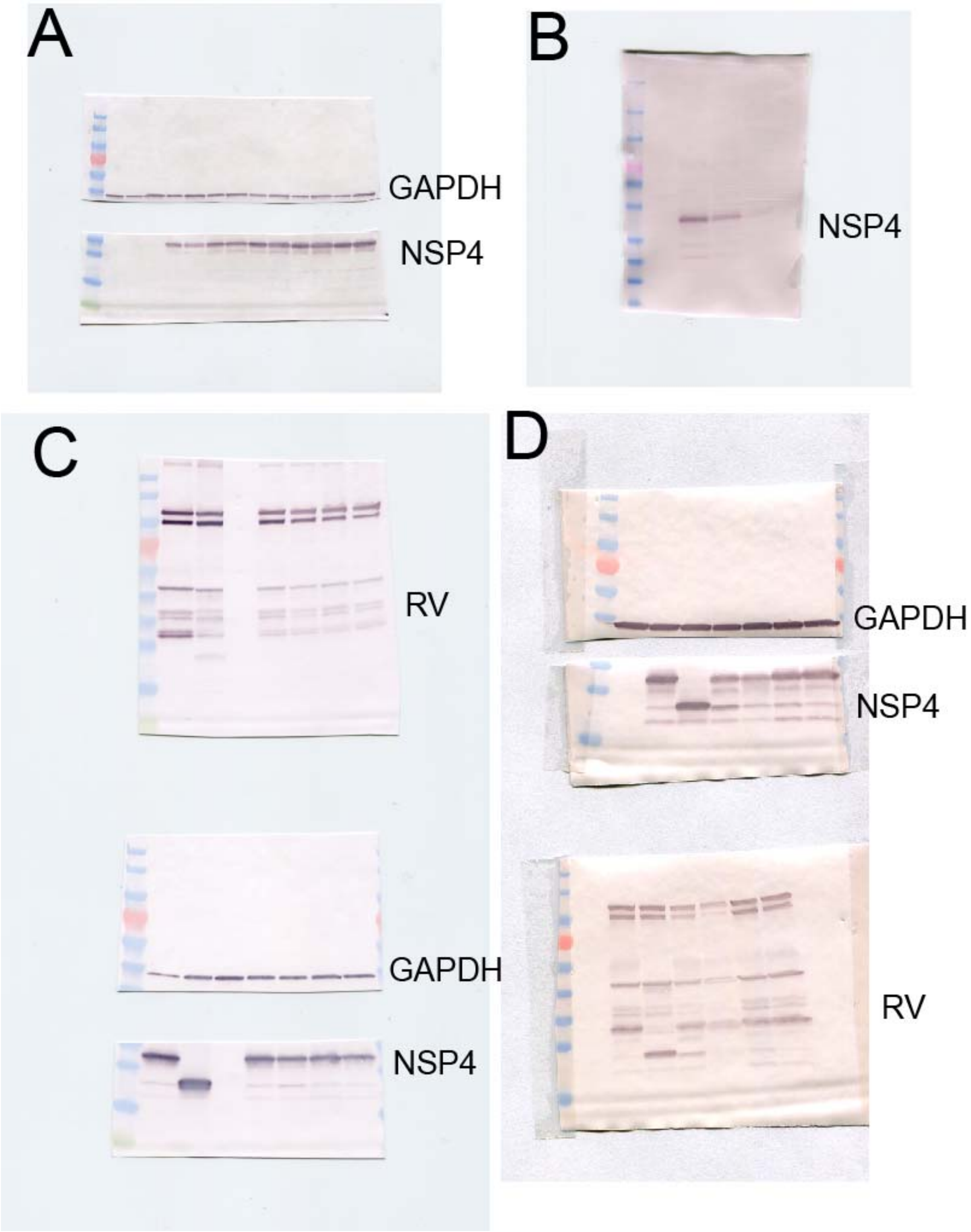

Supplementary Figure 2. Full-length images of immunoblots. **A.** Immunoblot corresponding to Fig 1B. The SDS-PAGE gel was transferred onto a single blot that was cut as shown. The upper portion was used to detect GAPDH and the lower portion was used to detect NSP4. **B.** Immunoblot corresponding to Fig. 5A. **C.** Immunoblots corresponding to Fig 8E-F. Two identically loaded SDS-PAGE gels were transferred onto blots. One was used to detect RV proteins (top). The other (bottom) was cut as shown. The upper portion was used to detect GAPDH and the lower portion was used to detect NSP4. **D.** Immunoblots corresponding to Fig 9F. Two identically loaded SDS-PAGE gels were transferred onto blots. One was used to detect RV proteins (top). The other (bottom) was cut as shown. The upper portion was used to detect GAPDH and the lower portion was used to detect NSP4.

Supplemental Video Descriptions

Supplemental Video 1

MA104-GCaMP5G cells were mock- or rotavirus (strain SA114F)-infected and live time-lapse imaging was performed. The video shows data from 3-14 hpi at 1 image/2 min. GCaMP5G reports cytoplasmic Ca^{2+} as changes in fluorescence intensity (green). Note the imbedded timer displays time from the beginning of acquisition not the time post-infection.

Supplemental Video 2

MA104-GCaMP5G cells were mock- or rotavirus (strain SA114F)-infected and live time-lapse imaging was performed from 6-7 hpi at 1 image/min. GCaMP5G reports cytoplasmic Ca^{2+} as changes in fluorescence intensity (green). After imaging, cells were fixed and stained for rotavirus antigen (red) and with DAPI to visualize the nuclei (blue). Immunofluorescence images were then superimposed to the time-lapse video. Note the imbedded timer displays time from the beginning of acquisition not the time post-infection.

Supplemental Video 3

MA104-GCaMP5G cells infected with SA114F (MOI 10) at ~7hpi were time-lapse imaged for ~10 min at 1 image/ 1.5 s. GCaMP5G reports cytoplasmic Ca^{2+} as changes in fluorescence intensity (green). After imaging, cells were fixed and stained for rotavirus antigen (red) and with DAPI to visualize the nuclei (blue). Immunofluorescence images were then superimposed to the time-lapse video. Note the imbedded timer displays time from the beginning of acquisition not the time post-infection.

Supplemental Video 4

MA104-GCaMP5G cells were mock-inoculated or rotavirus-infected with the recombinant SA11cl3-mRuby3 reporter virus and live time-lapse imaging was performed. The video shows data from ~2-18 hpi at 1 image/ 2 min. GCaMP5G reports cytoplasmic Ca^{2+} as changes in fluorescence intensity (green) and rotavirus protein synthesis is reported by mRuby3 expression (red) from the nonstructural protein 3 (NSP3) open-reading frame. Note the imbedded timer displays time from the beginning of acquisition not the time post-infection.

Supplemental Video 5

MA104-GCaMP5G cells were mock-inoculated or rotavirus-infected with porcine OSUa or OSUv strains (MOI 1) and live time-lapse imaging was performed. The video shows data from ~3-9 hpi at 1 image/min. GCaMP5G reports cytoplasmic Ca^{2+} as changes in

fluorescence intensity (green). Note the imbedded timer displays time from the beginning of acquisition not the time post-infection.

Supplemental Video 6

MA104-RGECO1.2/GCEPIAer cells were mock-inoculated or rotavirus-infected with SA114F (MOI 1) and live time-lapse imaging was performed. The video shows data from ~2-14 hpi at 1 image/2 min. RGECO1.2 reports cytoplasmic Ca^{2+} as increases in red fluorescence intensity and GCEPIAer reports endoplasmic reticulum Ca^{2+} as decreases in green fluorescence. Note the imbedded timer displays time from the beginning of acquisition not the time post-infection.

Supplemental Video 7

MA104-RGECO1.2/GCEPIAer cells were infected with SA114F (MOI 1) and live time-lapse imaging was performed. The video shows data for GECPIAer from ~2-15 hpi at 1 image/30 sec. The endoplasmic reticulum (ER) Ca^{2+} sensor GCEPIAer reports Ca^{2+} levels as changes in green fluorescence. Membrane compartments associated with rotavirus viroplasms are observed subsequent to the decrease in ER calcium levels. Note the imbedded timer displays time from the beginning of acquisition not the time post-infection.

Supplemental Video 8

MA104-GCaMP5G cells were mock-inoculated or rotavirus-infected with SA114F (MOI 1), then treated with vehicle control (DMSO), 50 μM 2-APB, or 10 μM BTP2 and live time-lapse imaging performed. The video shows data from ~2-19 hpi at 1 image/min. GCaMP5G reports cytoplasmic Ca^{2+} as changes in fluorescence intensity (green). Note the imbedded timer displays time from the beginning of acquisition not the time post-infection.

Supplemental Video 9

3D jejunum HIE-GCaMP6s enteroids were mock- or rotavirus (strain Ito)-infected and imaged once per 2 min in GFP and phase contrast on a widefield epifluorescence microscope for 6-22 hpi. GCaMP5G reports cytoplasmic Ca^{2+} as changes in fluorescence intensity (green). Note the imbedded timer displays time from the beginning of acquisition not the time post-infection.

Supplemental Video 10

Jejunum HIE-GCaMP6s enteroid monolayers were mock- or rotavirus (strain Ito)-infected, and treated with vehicle (DMSO) or 50μM 2-APB and imaged once per minute on a widefield epifluorescence microscope for 8-19 hpi. GCaMP5G reports cytoplasmic Ca^{2+} as changes in fluorescence intensity (green). Note the imbedded timer displays time from the beginning of acquisition not the time post-infection.
